# Cross-cultural development of an item list for computer-adaptive testing of fatigue in oncological patients

**DOI:** 10.1186/1477-7525-9-19

**Published:** 2011-03-29

**Authors:** Johannes M Giesinger, Morten Aa Petersen, Mogens Groenvold, Neil K Aaronson, Juan I Arraras, Thierry Conroy, Eva M Gamper, Georg Kemmler, Madeleine T King, Anne S Oberguggenberger, Galina Velikova, Teresa Young, Bernhard Holzner

**Affiliations:** 1 Department of Psychiatry and Psychotherapy, Innsbruck Medical University, Anichstr.35, A-6020 Innsbruck, Austria; 2 Department of Palliative Medicine, Bispebjerg Hospital, Bispebjerg bakke 23, DK-2400 Copenhagen, Denmark; 3 Division of Psychosocial Research & Epidemiology, Cancer Institute, Plesmanlaan 121, 1066 CX Amsterdam, The Netherlands; 4 Medical Oncology Department, Hospital of Navarre, C/Irunlarrea 3, ES-31008 Pamplona, Spain; 5 Medical Oncology Department, Centre Alexis Vautrin, 6 Avenue de Bourgogne, F-54500 Vandoeuvre-lès-Nancy, France; 6 School of Psychology, University of Sydney, Brennan MacCallum Building A18, AU-2006 Sydney, Australia; 7 Cancer Research UK Centre, University of Leeds, Leeds, UK; 8 Lynda Jackson Macmillan Centre, Mount Vernon Cancer Centre, Rickmansworth Rd, GB-HA62RN Northwood, UK

## Abstract

**Introduction:**

Within an ongoing project of the EORTC Quality of Life Group, we are developing computerized adaptive test (CAT) measures for the QLQ-C30 scales. These new CAT measures are conceptualised to reflect the same constructs as the QLQ-C30 scales. Accordingly, the Fatigue-CAT is intended to capture physical and general fatigue.

**Methods:**

The EORTC approach to CAT development comprises four phases (literature search, operationalisation, pre-testing, and field testing). Phases I-III are described in detail in this paper. A literature search for fatigue items was performed in major medical databases. After refinement through several expert panels, the remaining items were used as the basis for adapting items and/or formulating new items fitting the EORTC item style. To obtain feedback from patients with cancer, these English items were translated into Danish, French, German, and Spanish and tested in the respective countries.

**Results:**

Based on the literature search a list containing 588 items was generated. After a comprehensive item selection procedure focusing on content, redundancy, item clarity and item difficulty a list of 44 fatigue items was generated. Patient interviews (n = 52) resulted in 12 revisions of wording and translations.

**Discussion:**

The item list developed in phases I-III will be further investigated within a field-testing phase (IV) to examine psychometric characteristics and to fit an item response theory model. The Fatigue CAT based on this item bank will provide scores that are backward-compatible to the original QLQ-C30 fatigue scale.

## 1 Introduction

Cancer-related fatigue is frequently understood to be the most common symptom associated with cancer and its treatment [1-3]. By reducing a patient's ability to engage in meaningful personal work and social activities, fatigue has a major negative impact upon quality of life (QOL) [4,5].

Although there is no consensus on the definition and some researchers suggest that there is no qualitative difference between cancer-related fatigue and the tiredness experienced by the general population [6], others consider the concept of cancer-related fatigue as a distinct entity [7-10].

Common features of cancer-related fatigue definitions given in the literature [6,8,9,11] are a feeling of continuous tiredness and lack of energy associated with the treatment or the tumour. Moreover, the fatigue level is considered inadequate for the activity level and fatigue is not reduced by rest or sleep.

In addition to this general definition of fatigue, common fatigue subdimensions found in the literature are emotional, physical and cognitive fatigue [7,12,13]. Physical fatigue is related to a lowered level of ability, a feeling of weakness and an increased need for rest and sleep. Emotional fatigue covers sadness, anxiety, and diminished motivation. Cognitive fatigue includes decreased concentration, difficulty to think coherently, and mental exhaustion [7].

In this context, general fatigue can be defined as fatigue without the emotional or cognitive aspects. But the concepts of general and physical fatigue are more difficult to differentiate from a rational point of view as well as empirically [12].

Currently, a range of paper-pencil-based assessment instruments for fatigue have been validated. These instruments are unidimensional or multidimensional and assess intensity and/or impact of fatigue [4,12-15]. In addition to these specific instruments, fatigue is also covered by the two major QOL instruments in oncology, the EORTC QLQ-C30 [16] and the FACIT measurement system [4,17].

In the US there are two major projects on the development of fatigue item banks. Lai et al. [18] developed an English item bank containing 72 fatigue items and showing good psychometric properties. This item bank is mainly based on the FACIT-F items and covers various aspects of fatigue (e.g. physical, social, mental fatigue). Despite some heterogeneity in content, the items fit a unidimensional measurement model [19].

In addition, the PROMIS project [20] is developing item banks for a range of major PROs, for use across multiple fields of medical research. Details on the PROMIS fatigue item bank are available via the PROMIS Assessment Center website [21].

The EORTC Quality of Life Group has been conducting an independent project to develop computer-adaptive versions of the QLQ-C30 scales [22,23]. Computer-adaptive testing (CAT) is an advanced method to assess patient-reported outcomes (PROs). With the help of an algorithm CAT selects individually tailored item sets from an item bank. It does so by estimating a patient's fatigue level after each response and then selecting the next most appropriate item for this fatigue level. To cover the fatigue continuum a comprehensive item bank containing items on various degrees of fatigue is necessary.

Taking a cross-cultural approach the EORTC project is developing CAT measures for several European languages simultaneously to guarantee wide applicability. This means that several collaborators from across Europe, and recently also Australia, are involved in all stages of the development process.

As the fatigue CAT is among the first measures to emerge from the EORTC CAT project, we would like to present the development of the fatigue item bank in detail to shed light on the EORTC approach to CAT development. Whereas Petersen et al. [22] have described the general methodology, this paper aims at exemplifying individual development steps. These details should make the process of item bank development transparent to future users of the EORTC Fatigue CAT.

In detail, the study described in this paper addressed the following aims:

• Literature search to set up a comprehensive fatigue item list

• Item selection and operationalisation

• Cross-cultural item pre-testing in cancer patients

• Construction of an item list for international field testing

## 2 Methods

An overview on the EORTC CAT development strategy is given by Petersen et al. [22]. In the main, it comprises four phases (literature search, operationalisation, pre-testing and field testing) resulting in an item bank for CAT. A major focus of the EORTC strategy is guaranteeing cross-cultural applicability of the CAT from the very beginning.

The very first step of CAT development was defining the fatigue concept that should be assessed with the new CAT. As pointed out above the newly developed fatigue CAT should assess the same concept as the fatigue scale of the QLQ-C30. Currently, the QLQ-C30 fatigue scale consists of only three items. In line with the fatigue definitions given above these items are considered to cover general fatigue ("Did you need to rest?", "Were you tired?") and physical fatigue ("Have you felt weak?"). The items use four response categories ("not at all" - "a little" - "a bit" - "very much") for assessing severity and intensity of these two fatigue aspects.

### Phase 1: Literature search

To set up an initial item list a literature search was performed focusing on items assessing fatigue in cancer patients. Abstracts or questionnaires published until August 2008 in one of the following databases were included in the search: PubMed http://www.pubmed.org, PROQOLID http://www.proqolid.org, Psyndex Tests http://www.ebscohost.com, and the EORTC Quality of Life Group item bank (covering all items used within EORTC questionnaires; http://www.eortc.be/itembank2. As search term, we used: (CANCER or NEOPLASMS or TUMO*R or CHEMOTHERAPY or ONCOL*) and (FATIGUE or TIREDNESS or DROWSINESS) and (QUESTIONNAIRE or INVENTORY or SCALE or MODULE or MEASURE*).

All items from questionnaires or subscales claiming to assess fatigue or a closely related construct were entered in an initial item list.

### Phase 2: Operationalisation

To obtain an item list for pre-testing in patients, the collected items underwent a comprehensive item selection procedure. At each of the following evaluation steps two reviewers evaluated the items independently and consequently discussed disagreements face-to-face to reach consensus. In complicated cases, discussion also included further researchers or literature.

1. The collected items were categorized as measuring either physical or general fatigue, or a different construct. Items considered as not measuring physical or general fatigue were discarded from the item list.

2. Items rated as redundant and items that could not be reformulated to fit the EORTC item style were removed from the item list. EORTC item style implies the following characteristics:

a. Item assesses symptom severity or intensity

b. Item uses the response format "not at all" - "a little" - "quite a bit" - "very much"

c. Item refers to the past week

d. Item is phrased in a way that "very much" indicates high symptom burden

e. If possible, item starts with "Did you..." or "Have you..."

3. Using the items selected in step 2 as inspiration, new items fitting the EORTC item style were formulated.

4. The items constructed in step 3 were evaluated with regard to redundancy and clarity.

5. To obtain a first impression of whether the remaining items cover the fatigue continuum sufficiently, all items were categorized as measuring mild, moderate or severe fatigue levels. This allowed for the generation of new items in case of insufficient coverage.

6. As a final step before pre-testing, several experts reviewed the remaining items. First, items and selection procedure were reviewed by two senior members of the EORTC QLG. Second, members of the EORTC CAT group evaluated the item list. Third, ten international experts in the field of fatigue assessment were asked to evaluate: what the items measure, how relevant they are for fatigue measurement, whether they are appropriate, and whether they are clear and well-formulated. Items considered problematic by at least three of the reviewers were discussed further and possibly revised or deleted. As experts participating in these evaluations were from different centres across Europe and Australia, discussion was mostly done via E-Mail.

### Phase 3: Pre-testing

To collect patient feedback, the items were translated into the languages of the participating centres by the EORTC Quality of Life Department. Items were translated from English into the target languages and then back-translated. Details on the translation process are given in the EORTC translation manual [24]. Ethical approval was obtained at local ethical committees of centers contributing patients.

The patient interviews helped to pre-test item wording (e.g. whether the items are confusing, intrusive, difficult, upsetting or annoying) and to find out whether relevant issues have been missed during the previous steps. Due to the number of items, questions were directed towards the entire item list rather than towards single items. Recommendations for patient interviews in the EORTC QLG guidelines for developing questionnaire modules were followed [25].

Comments on the following issues were not included in the analysis as they were not relevant to the aims of this project:

• Similarity of items: This is inherent to the development of an item bank for CAT aiming at covering the whole continuum of fatigue

• Response format: As the project aimed at developing CAT for the QLQ-C30 the response format was pre-determined and was not to be revised.

• Lacking assessment of other fatigue aspects: Again, the CAT aimed at measuring the same construct as the QLQ-C30, i.e. general and physical fatigue.

• Positive comments (e.g. on the importance of fatigue assessment in general)

## 3 Results

### Phase 1: Literature search

The literature search resulted in 37 fatigue assessment instruments and fatigue subscales within QOL instruments (see Table 1) containing 588 items.

**Table 1:** Fatigue assessment instruments collected from literature search (Phase I)

#	Acronym	Full name	Reference
1.	BFI	Brief Fatigue Inventory	[15]
2.	CFQ	Chalder Fatigue Questionnaire	[28]
3.	CFS	Cancer Fatigue Scale	[29]
4.	CFS	Chalder Fatigue Scale	[28]
5.	CRFDS	Cancer-Related Fatigue Distress Scale	[30]
6.	DEFS	Dutch Exertion Fatigue Scale	[31]
7.	D-FIS	Daily Fatigue Impact Scale	[32]
8.	DUFS	Dutch Fatigue Scale	[31]
9.	EORTC QLQ-C30	Quality of Life Questionnaire - Core 30	[16]
10.	EORTC QLQ-HDC29	Quality of Life Questionnaire - High-Dose Chemotherapy 29	[33]
11.	EORTC QLQ-MY20	Quality of Life Questionnaire - Multiple Myeloma 20	[34]
12.	EORTC QLQ-OV28	Quality of Life Questionnaire - Ovarian 28	[35]
13.	EORTC QLQ-FA13	Quality of Life Questionnaire - Fatigue 13	[13]
14.	FACT-F/An	Functional Assessment of Cancer Therapy - Fatigue/Anemia	[4]
15.	FAI	Fatigue Assessment Instrument	[36]
16.	FAQ	Fatigue Assessment Questionnaire	[37]
17.	FAS	Fatigue Assessment Scale	[38]
18.	EORTC QLQ-FA	EORTC Fatigue Module Phase (Development phase II)	[13]
19.	FDS	Fatigue Descriptive Scale	[39]
20.	FIS	Fatigue Impact Scale	[40]
21.	FSCL	Fatigue Symptoms Checklist	[41]
22.	FSI	Fatigue Symptom Inventory	[42]
23.	FSS	Fatigue Severity Scale	[43]
24.	IFS	Iowa Fatigue Scale	[44]
25.	LFS	Lee Fatigue Scale	[11]
26.	MAF	Multidimensional Assessment of Fatigue	[45]
27.	MFI	Multidimensional Fatigue Inventory	[12]
28.	MFIS	Modified Fatigue Impact Scale	[46]
29.	MFSI	Multidimensional Fatigue Symptom Inventory	[47]
30.	MFSI-SF	Multidimensional Fatigue Symptom Inventory - Short Form	[48]
31.	PFS	Piper Fatigue Scale	[49]
32.	SCFS-6	Schwartz Cancer Fatigue Scale	[50]
33.	SFS	Situational Fatigue Scale	[51]
34.	SOFA	Schedule of Fatigue and Anergia	[52]
35.	SOF	Swedish Occupational Fatigue Inventory	[53]
36.	WCFS	Wu Cancer Fatigue Scale	[54]
37.	WEIMuS	Würzburger Erschöpfungsinventar für Multiple Sklerose	[55]

### Phase 2: Operationalisation

#### Step 1: Item classification

Each of the 588 items was classified to either physical fatigue (88 items), general fatigue (258 items), or as measuring another construct (242 items). The reviewers agreed on the classification of 80% of the items, on 20% they disagreed and reached a consensus choice after discussion. Examples for disagreement are: "I am not interested in sex" (no fatigue vs general fatigue → no fatigue: item considered as too unspecific), or "I feel slowed down" (cognitive fatigue vs general fatigue → general fatigue: slowed down was considered to also be related to physical aspects of fatigue). After this step the database included 346 items

#### Step 2: EORTC item style and redundancy

To facilitate the detection of redundant items in this large item set, all items were first classified into ad-hoc categories (e.g. physical, social, household, energy) that were set up by the reviewers.

With regard to item in- or exclusion reviewer agreement was 88%. As the final item list for pre-testing in patients should not be too long, in addition to discarding items that met the strict redundancy criteria, others were discarded because they were very similar in meaning (e.g. *"I get little done" *and *"I think I do very little in a day", or "I don't do much during the day" *and *"I do quite a lot within a day")*. For each group of "duplicate" items, the item judged by the two reviewers to be the best in terms of clarity and proximity to EORTC item style was retained.

The two reviewers agreed that 145 items were redundant or duplicates of other better items, and were therefore deleted. A further 54 items were excluded because they did not fit EORTC item style and could not be rephrased to do so (e.g. "*The fatigue or tiredness I am having causes me distress because it: makes me feel totally exhausted" *does not assess fatigue severity; *"Rate how much of the day, on average, you felt fatigued in the past week" *could not be rephrased to fit response format). After this selection step 147 items remained.

#### Step 3: Item reformulation

The 147 remaining items were reformulated to fit the EORTC item style. For example, based on the item *"I didn't have the energy to get up and do things" *the new item *"Did you lack the energy to get up and do things?" *was formulated. As the suitability for reformulation into the EORTC item style already had been assessed in step two, no items were deleted in this step.

#### Step 4: Item clarity and redundancy

**Figure 1:**
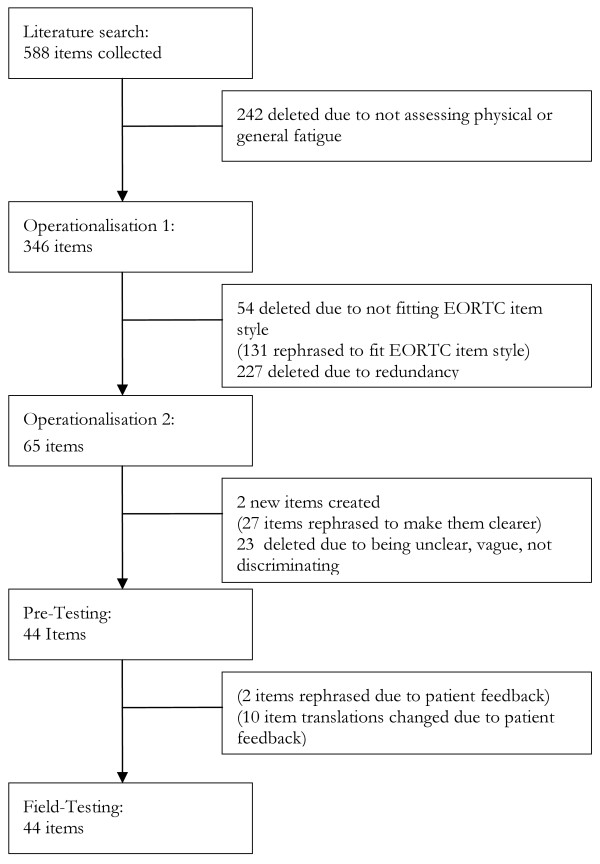
**Item selection procedure**.

This step repeats the redundancy evaluation in step two, but now for the newly reformulated items fitting the EORTC item style. Items that duplicated other items were deleted (reviewer agreement 74%). This step resulted in a reduction of the list to 65 items. For a summary see Figure 1: Operationalisation 1.

#### Step 5: Coverage of the fatigue continuum

Each of the remaining 65 items was rated with regard to its relevance for patients with mild, moderate or severe fatigue levels (reviewer agreement 55%). 22 items were classified as measuring mild fatigue (e.g. *"Did you get fatigued from exercising?"*), 27 were rated as mostly relevant for patients with moderate fatigue problems (e.g. *"Did you become easily tired?"*) and 16 were judged mostly relevant for patients with severe fatigue (e.g. *"Did you find it fatiguing to stand under the shower?"*). This indicated sufficient coverage so the creation of new items was not required.

#### Step 6: Expert reviews

According to suggestions by the reviewers within the EORTC Quality of Life Group, we avoided the term "fatigued" as the translation into other languages may be difficult. Its meaning might be captured best with the terms "tired" or "exhausted". Items were rephrased accordingly (two items were rephrased using "tired" as well as "exhausted", i.e. two new items were generated). 14 items were deleted due to redundancy after rephrasing.

As part of the further review procedure we included revisions requested by the EORTC CAT Group. Four items were reformulated due to unclear wording, one item was deleted due to problems concerning the translation into other languages (*"Have you felt heavy"*) and one item was deleted because it was considered as not measuring fatigue (*"Have you felt lazy?"*).

Final item evaluation was done by 10 international experts (5 physicians and 5 psychologists) from the following countries: Denmark (3), Austria (3), Australia (2), Italy (1), and Germany (1). These experts were asked to evaluate: what the items measure, how relevant they are for measurement of FA, whether they are appropriate, and whether they are clear and well-formulated. Consequently, two items were rephrased and seven items were excluded for being too ambiguous, too vague or not being able to distinguish between patients with low and high fatigue levels (e.g. *"Have you felt inactive?" *or *"Have you found participation in family activities exhausting?"*).

Thus, the final list for pre-testing in patients comprised 44 items (25 items for general fatigue and 19 items for physical fatigue). For a summary see Figure 1: Operationalisation 2.

### Phase 3: Pre-testing

For collecting patient feedback the English item list was translated to Danish, French, German, and Spanish. A total of 52 patients at five centres (in Austria, Denmark, France, Spain, and the UK) completed the 44 items and commented on them. For details on patient characteristics see Table 2.

**Table 2:** Descriptive statistics for the patient feedback sample (phase III)

Language	Danish	23.1%
	English	19.2%
	French	19.2%
	German	19.2%
	Spanish	19.2%
Age (years)	Mean (range)	57.4 (32-80)

Sex	Women	56.9%
	Men	43.1%

Marital status	Partnership, marriage	84.0%
	Living alone	16.0%

Education	<10 years	21.6%
	11-13 years	43.1%
	14-16 years	15.7%
	>16 years	19.6%

Employment status	Full time	19.6%
	Part time	17.6%
	Unemployed	3.9%
	Retired	43.1%
	Other	15.7%

Tumor type	Breast cancer	26.9%
	Lung cancer	19.2%
	Colorectal cancer	15.4%
	Gynaecological cancer	9.6%
	Laryngeal/Pharyngeal cancer	5.8%
	Bladder cancer	5.8%
	Other	17.3%

Tumour stage	Local/Locoregional (I, II)	33.3%
	Advanced (III, IV)	64.7%
	Unknown	2.0%

Current treatments*	No current treatment	13.5%
	Chemotherapy	65.4%
	Radiotherapy	21.2%
	Surgery	7.7%
	Endocrine therapy	5.8%
	Other	11.5%

*multiple treatments per patients possible

#### Translation issues

The EORTC Quality of Life Department Translation Office translated the item list into the languages of the participating centres. Researchers at the participating centres then reviewed the item list for their respective first language and suggested necessary changes.

Based on patient feedback (see below), translations of three Danish, two German and five French items were corrected. The English and Spanish version did not require changes.

#### Patient feedback on items

Six patients regarded the term "exhausted" as being too strong or confusing and suggested terms referring to a lesser degree of fatigue. Since items assessing severe fatigue are necessary for the final CAT, no changes were made regarding this.

Several items were commented as being too unspecific, i.e. asking too broadly for a certain aspect of fatigue (e.g. *"Have you been so tired it was difficult keeping your eyes open?"*). Problems were identified with the use of the word 'things' which was considered unspecific. (e.g. *"Have you lacked the energy to do things?"*). A number of these broad items were rated as difficult or confusing.

Items rated as annoying were mostly those that were only slightly different from other items, thus appearing as unnecessary and repetitive. No items were found to be intrusive or upsetting.

Based on patient feedback two items were changed to be more specific (e.g. *"Have you been so tired it was difficult keeping your eyes open *during daytime?"). With regard to the term "things" no changes were made as a replacement of the word with a description of an activity was considered to limit the applicability of the items considerably.

#### Further items suggested by patients

To investigate fatigue coverage of the item list, patients were asked to suggest additional items. Examples of issues raised by patients were feeling tired when using public transport (e.g. waiting for buses, or a free seat), fatigue in situations not within daily life (e.g. travelling, going to the theatre), or impact of fatigue on the ability to do one's job

As these issues were not within the scope of the intended fatigue CAT or were considered not to be applicable to the majority of patients, it was decided within the CAT group not to add further items to the item list.

The final item list comprising 44 items for field testing within phase IV is shown in Table 3.

**Table 3:** Item list for field testing in phase IV

#	Item text
Item 01	Have you found talking exhausting?
Item 02	Have you been so tired it was difficult keeping your eyes open during daytime?
Item 03	Have your muscles felt very tired after physical activity like taking a long walk?
Item 04	Have you woken up with a feeling of exhaustion?
Item 05	Have you started things without difficulty but got weak as you went on?
Item 06	Have you lacked the energy to do things?
Item 07	Have you needed to lie down during the day?
Item 08	Have you felt slowed down?
Item 09	Have you been too tired to do your usual activities?
Item 10	Have you felt drained?
Item 11	Have you been so exhausted it felt almost impossible to move your body?
Item 12	Have you had trouble starting things because you were tired?
Item 13	Have you been too tired to do even simple things?
Item 14	Have you found shopping and doing errands exhausting?
Item 15	Have you felt sleepy during the day?
Item 16	Have you felt physically exhausted?
Item 17	Have you found leisure and recreational activities exhausting?
Item 18	Have you felt weak in your arms or legs?
Item 19	Have you felt exhausted?
Item 20*	Were you tired?
Item 21	Have you slept during the day?
Item 22	Have you had to sleep for long periods during daytime?
Item 23	Have you lacked energy?
Item 24	Have you become easily tired?
Item 25	Have you become tired from dressing?
Item 26	Have you had trouble sitting up because you were tired?
Item 27*	Have you felt weak?
Item 28	Have you felt worn out?
Item 29	Have you felt like falling asleep during the day?
Item 30	Have you had a feeling of overwhelming and prolonged lack of energy?
Item 31	Have you become tired from taking a shower?
Item 32	Have you had trouble finishing things because you were tired?
Item 33	Have you become tired from walking up stairs?
Item 34	Have you become tired from washing yourself?
Item 35	Have you become tired from taking a short walk?
Item 36*	Did you need to rest?
Item 37	Have you required frequent or long periods of rest?
Item 38	Have you been too tired to eat?
Item 39	Have you become tired from carrying out your duties and responsibilities?
Item 40	Have you found physical activities, like taking a long walk, exhausting?
Item 41	Have you had an extreme need for rest?
Item 42	Have you become exhausted from dressing?
Item 43	Have you felt tired for a long time after physical activity like taking a long walk?
Item 44	Have you become exhausted from taking a shower?

*item from the EORTC QLQ-C30 fatigue scale

## 4 Discussion

The main objective of this study was to develop an item bank for computer-adaptive testing of the fatigue concept currently covered by EORTC QLQ-C30 Fatigue scale. This item bank should cover the same aspects of fatigue as the QLQ-C30, i.e. general and physical fatigue.

The extensive literature search and the multi-step item selection through reviews by experts in the field as well as through cross-cultural patient feedback interviews resulted in an elaborate item list for the assessment of fatigue in cancer patients. These 44 items are currently available in five European languages and will be further investigated with regard to psychometric properties in phase IV of the EORTC CAT development process. The whole development process was defined based on the EORTC approach to module development.

The predefined item selection criteria concerning content and scope as well as the specific sequence of selection steps described above aimed to make the development process as transparent as possible. The inclusion of experts from different fields and of patients in the item list construction were important to guarantee content coverage and item suitability.

Whilst patient feedback is important to validate translations and assess coverage, several issues raised by patients could not be incorporated into the item list, e.g. issues relating to aspects of fatigue not covered by the EORTC QLQ-C30 Fatigue scale.

The restriction of the CAT to cover only physical and general fatigue in order to replicate the QLQ-C30 fatigue scale narrows the coverage of fatigue. In addition, the use of a specific item format (EORTC item style) further narrowed the item list. But these restrictions also guarantee backward-compatibility with QLQ-C30 data collected within numerous studies. The latter allows comparison of scores derived from CAT to scores derived from the original QLQ-C30. The three original fatigue items from the EORTC QLQ-C30 are also part of the new item bank. This relates the CAT to a huge amount of data from patients from different countries, with different diagnoses, during different treatment phases, and receiving different treatments.

Thus, it will combine the advantages of a familiar instrument and extensive reference data with the obvious advantages of CAT, i.e. a relatively low number of items providing high measurement precision. The short assessment time is of particular importance in fatigued patients, to whom filling in long questionnaires poses a significant burden. This burden may result in selection bias as severely fatigued patients are likely to be excluded in traditional patient-reported outcome assessments.

Another characteristic the EORTC Fatigue CAT shares with the QLQ-C30 is that fatigue is considered to be a "quasi-trait" according to Reise and Waller [26]. This means it is a unipolar construct where the opposite of fatigue is the absence of fatigue and not, for example, being full of energy. Whilst this is a reasonable approach to defining health outcomes in oncological patients, it might limit applicability to the general population as floor effects are likely to occur. However, constructing a bipolar scale including both positive items (e.g. feeling energetic) and negative items (e.g. feeling tired) may impair item homogeneity and result in a two dimensional structure. It should be noted, that the EORTC fatigue item bank is not only usable for CAT applications but also for the development of IRT-based static short forms, i.e. fixed item sets applicable as paper-pencil questionnaires.

As mentioned previously the major US-initiative PROMIS supported by the NIH is developing item banks for major PROs. Within this comprehensive project a fatigue item bank was developed to enable CAT and the creation of static short forms. Compared to the broad fatigue item bank of PROMIS (covering e.g. physical and mental fatigue, frequency and severity of fatigue, and the opposite of fatigue, i.e. feeling energetic), the EORTC fatigue item bank is narrower focusing only on severity and intensity of general and physical fatigue. Also, the EORTC project has a strong focus on cross-cultural applicability of the item bank and consequently includes collaborators and patients from various countries in all development stages. In contrast, development of the PROMIS item banks is done in English only, although future translations are intended [21]. To guarantee these translations PROMIS employs expert ratings on ease of translation [27].

In addition to the EORTC CAT project, the EORTC Quality of Life Group is continuing to develop modules to supplement the QLQ-C30 with regard to certain patient groups or specific issues. For the multidimensional assessment of fatigue a questionnaire module (EORTC QLQ-FA13) is currently under development [13]. It has been pre-tested in about 300 patients and six different languages and will assess physical, cognitive, and emotional fatigue as a traditional paper-pencil measure. By developing a backward-compatible CAT measure as well as a multidimensional questionnaire module, the EORTC measurement system extends its scope in two directions. On the one hand the Fatigue CAT will provide an improved measure for the generic QLQ-C30 fatigue scale; on the other hand the QLQ-FA13 will be a measure for assessing specific subdimensions of fatigue.

The next step in this EORTC project (phase IV) will determine psychometric item characteristics based on a large patient sample recruited from oncological centres in Australia, Austria, Denmark, France, Germany, the Netherlands, Spain and the UK. The cross-cultural patient recruitment will allow for detailed analyses of differential item functioning with regard to culture/language.

Successful implementation of CAT into clinical routine and trials requires adequate software solutions for item administration. Such software packages have to include as a minimum, a CAT algorithm for item selection, the item bank with psychometric characteristic and a patient-interface presenting items graphically and collecting responses. In addition to these basic features, ideal software should provide data storage and elaborate graphical presentation of results to medical staff. Over the last few years software development for electronic patient-reported outcome assessment including CAT administration has been given increasing attention within the EORTC Quality of Life group and software for CAT administration is being developed in parallel with the item pool development.

## Conflict of interests

The authors declare that they have no competing interests.

## Authors' contributions

JMG, MAP, MG and BH participated in study design and coordination. JMG, EMG, GK, TC and AO performed the literature search for phase 1. JMG, MAP, MG, NKA, JIA, TC, EMG, GK, MTK, AO, GV, TY and BH were involved in the item selection procedure (phase 2 and 3). JMG, MAP, MG, and GK were involved in data analysis. JMG, MAP, MG, GK and BH helped to draft the manuscript. All authors read and approved the final manuscript.
